# Histone Deacetylase TaHDT701 Functions in TaHDA6-TaHOS15 Complex to Regulate Wheat Defense Responses to *Blumeria graminis* f.sp. *tritici*

**DOI:** 10.3390/ijms21072640

**Published:** 2020-04-10

**Authors:** Pengfei Zhi, Lingyao Kong, Jiao Liu, Xiaona Zhang, Xiaoyu Wang, Haoyu Li, Maokai Sun, Yan Li, Cheng Chang

**Affiliations:** 1 College of Life Sciences, Qingdao University, Qingdao 266071, China; 2 College of Life Sciences, Capital Normal University, Beijing 100084, China

**Keywords:** HDT701, HDA6, histone modification, wheat, *Blumeria graminis* f.sp. *tritici*

## Abstract

Powdery mildew disease caused by *Blumeria graminis* f.sp. *tritici* (*Bgt*) leads to severe economic losses in bread wheat (*Triticum aestivum* L.). To date, only a few epigenetic modulators have been revealed to regulate wheat powdery mildew resistance. In this study, the histone deacetylase 2 (HD2) type histone deacetylase TaHDT701 was identified as a negative regulator of wheat defense responses to *Bgt*. Using multiple approaches, we demonstrated that TaHDT701 associates with the RPD3 type histone deacetylase TaHDA6 and the WD40-repeat protein TaHOS15 to constitute a histone deacetylase complex, in which TaHDT701 could stabilize the TaHDA6-TaHOS15 association. Furthermore, knockdown of *TaHDT701*, *TaHDA6*, and *TaHOS15* resulted in enhanced wheat powdery mildew resistance, suggesting that the TaHDT701-TaHDA6-TaHOS15 histone deacetylase complex negatively regulates wheat defense responses to *Bgt*. Moreover, chromatin immunoprecipitation assays revealed that TaHDT701 could function in concert with TaHOS15 to recruit TaHDA6 to the promoters of defense-related genes such as *TaPR1*, *TaPR2*, *TaPR5*, and *TaWRKY45.* In addition, silencing of *TaHDT701*, *TaHDA6*, and *TaHOS15* resulted in the up-regulation of *TaPR1*, *TaPR2*, *TaPR5*, and *TaWRKY45* accompanied with increased histone acetylation and methylation, as well as reduced nucleosome occupancy, at their promoters, suggesting that the TaHDT701-TaHDA6-TaHOS15 histone deacetylase complex suppresses wheat powdery mildew resistance by modulating chromatin state at defense-related genes.

## 1. Introduction

*Blumeria graminis* f.sp. *tritici* (*Bgt*) is the obligate biotrophic fungal pathogen that is capable of infecting the important crop bread wheat (*Triticum aestivum* L.). Upon landing on the aerial surface of wheat, *Bgt* conidia first germinate and then penetrate the host cell wall to develop an intracellular feeding structure called haustorium to acquire both nutrients and water from host plant cells [1,2,3]. After successful colonization and proliferation, *Bgt* fungi finally form microcolonies and disperse conidia to infect more plants. As a detrimental disease caused by *Bgt*, powdery mildew disease usually leads to wheat yield losses of 10% to 40% [4,5,6,7,8]. Breeding resistant wheat varieties is a safe and effective approach to control this disease. To this end, it is vital to identify the wheat genes regulating the plant defense responses to *Bgt* and unravel their underlying mechanisms.

Pathogen recognition by plants results in the activation of plant defense reactions, which are usually accompanied by global transcriptional reprogramming to favor plant defense responses over normal cellular functions [9,10]. Therefore, defense-related gene expression and the consequent defense responses in the plant are tightly regulated, permitting the plant to respond promptly to the pathogen attack [11,12,13]. There is growing evidence indicating that plant defense-related gene expression is fine-tuned by the epigenetic mechanisms such as the histone post-translational modifications, which usually influences the access of DNA to the transcriptional machinery [12,14,15]. For instance, acetylation of histone lysine residues generally perturbs the interactions between nucleosomes, leading to a looser chromatin state more permissive for transcription [16,17].

Histone deacetylases (HDACs) catalyze the histone deacetylation and remove the acetyl group from histones. Plant HDACs can be divided into four major families, including the HDA1 (histone deacetylase 1 protein)-like family, the SIR2 (silent information regulator protein 2)-like family, the RPD3 (reduced potassium dependency protein 3)-like family and the plant-specific histone deacetylase 2 (HD2) family [18,19]. Increasing evidence has revealed that plant HDACs usually act as components of multiprotein complexes to modulate transcription during various biological processes. For example, the RPD3 type deacetylase AtHDA19 interacts with the WD40-repeat (WDR) protein AtMS1 to form an HDAC complex that fine-tunes ABA signaling in *Arabidopsis thaliana* [20]. Furthermore, the Arabidopsis RPD3 type deacetylase AtHDA6 interacts with the HD2 type deacetylase AtHD2C to regulate the gene expression involved in the ABA and NaCl responses [21]. More recently, AtHD2C was reported to interact with the WDR protein AtHOS15, thereby modulating chromatin status and gene expression of cold-responsive COR genes in response to freezing stress in Arabidopsis [22].

In recent years, many histone deacetylases have been described as positive or negative regulators of plant defense responses [12,14,15]. For example, Arabidopsis RPD3 type histone deacetylases AtHDA19 and AtHDA6 were reported to positively regulate the jasmonic acid (JA)-mediated defense signaling, while suppressing the salicylic acid (SA)-mediated defense responses against the biotrophic pathogens [23,24,25,26]. Similarly, the Arabidopsis homolog of the yeast Sir2 deacetylase, AtSRT2, negatively regulates the plant defense to *Pseudomonas syringae* pv *tomato* DC3000 (*Pst* DC3000), possibly by suppressing the expression of SA biosynthesis-related genes [27]. In addition, the HD2 type deacetylase AtHD2B was reported to be activated by MPK3-mediated phosphorylation and could mobilize from the nucleolus to the nucleoplasm to fine-tune the defense responses to *Pst* DC3000 [28]. Similar studies have been described in crop plants. For instance, the wheat RPD3 type deacetylase TaHDA6 was recently reported to interact with the WDR protein TaHOS15 to suppress the gene expression of defense-related genes by histone deacetylation, thereby negatively regulating the defense responses to *Bgt* in bread wheat [29].

A previous study revealed that the rice HD2 type histone deacetylase OsHDT701 negatively regulates rice defense responses to pathogens *Magnaporthe oryzae* (*M. oryzae*) and *Xanthomonas oryzae* pv *oryzae* (*Xoo*) by reducing the levels of histone H4 acetylation at defense-related genes [18]. However, the biological function of HDT701 in other crop plants such as bread wheat remains to be uncovered. In this study, we characterized the function of wheat TaHDT701 in regulating the defense responses to *Bgt* and found that TaHDT701 interacted with the RPD3 type deacetylase TaHDA6 and the WDR protein TaHOS15 to constitute a histone deacetylase complex and the TaHDT701-TaHDA6-TaHOS15 histone deacetylase complex negatively regulates the wheat defense responses to powdery mildew by modulating the chromatin state at defense-related genes.

## 2. Results

### 2.1. TaHDT701 Negatively Regulates The Wheat Defense Responses to Powdery Mildew

To characterize the function of bread wheat HD2 type histone deacetylase TaHDT701 in regulating the wheat defense responses to powdery mildew, we first identified the wheat TaHDT701 based on the protein sequence of rice OsHDT701(Os05g51830) and the genome sequence of hexaploid bread wheat (*Triticum aestivum* L.) [30]. Three highly homologous sequences of wheat *TaHDT701* were isolated from wheat cultivar Yannong 999. Through searching the chromosome-based draft sequences of *T. aestivum* [30], we mapped these *TaHDT701* sequences on the chromosomes 1A, 1B, and 1D, which were designated as *TaHDT701-A*, *TaHDT701-B*, and *TaHDT701-D*. Sequence alignment demonstrated that the coding regions of allelic *TaHDT701-A*, *TaHDT701-B*, and *TaHDT701-D* exhibited above 97% nucleotide sequence identity (Figure S1). The predicted TaHDT701-A, TaHDT701-B, and TaHDT701-D proteins had more than 51% amino acid sequence identities with HvHDT701 from *Hordeum vulgare*, BdHDT701 from *Brachypodium distachyon*, OsHDT701 from *Oryza sativa*, and AtHD2A, AtHD2B, and AtHD2C from *Arabidopsis thaliana* (Figure S2).

**Figure 1:**
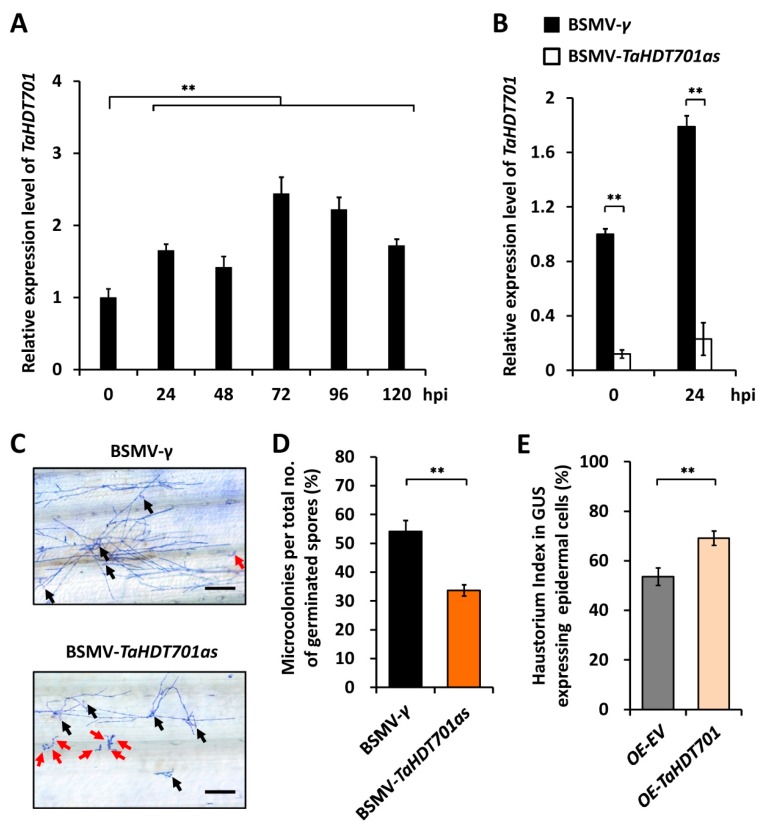
*TaHDT701* is a negative regulator of bread wheat resistance to the powdery mildew pathogen *Blumeria graminis* f.sp. *tritici* (*Bgt*). (**A**) Expression profiles of *TaHDT701* in Yannong 999 leaves during the infection of *Bgt* virulent isolate E09. (**B**) Transcript abundance of *TaHDT701* in Yannong 999 leaves inoculated with barley stripe mosaic virus (BSMV)-*γ* and BSMV-*TaHDT701as.* (**C**) *Bgt* microcolony formation type on wheat plants infected with BSMV-*γ* and BSMV-*TaHDT701as.* Conidia that germinated and finally established microcolonies are indicated with black arrows, and conidia that germinated but failed to establish microcolonies are indicated with red arrows. Bar, 150 μm. (**D**) Statistical analysis of *Bgt* microcolony formation on BSMV-*γ* and BSMV-*TaHDT701as* wheat leaves. For each treatment, at least 500 *Bgt-*wheat interaction sites were separately counted. (**E**) Statistical analysis of *Bgt* haustorial formation in wheat epidermal cells bombarded with OE-*EV* or OE-*TaHDT701*. At least 50 cells were analyzed in one experiment. For (**A**), (**B**), (**D**), and (**E**), three independent biological replicates per treatment were statistically analyzed (t-test, ** *p* < 0.01).

Quantitative reverse transcription-polymerase chain reaction assay (qRT-PCR) revealed that the total transcript levels of all the endogenous *TaHDT701* genes, including *TaHDT701-A*, *TaHDT701-B*, and *TaHDT701-D,* marginally increased from 24 h to 120 h post inoculation (hpi) with conidia of *Bgt* virulent isolate E09 in the Yannong 999 seedlings (Figure 1A). Through employing the barley stripe mosaic virus (BSMV)-induced gene-silencing (BSMV-VIGS) system, we silenced all the endogenous *TaHDT701* genes in the Yannong 999 seedlings. qRT-PCR confirmed that the transcript level of *TaHDT701* was 8-fold lower in the BSMV-*TaHDT701as* plants compared with the BSMV-*γ* plants (Figure 1B). Thereafter, we inoculated these BSMV-VIGS wheat plants with conidia of *Bgt* virulent isolate E09 and analyzed the formation of *Bgt* microcolony (Figure 1C). As shown in Figure 1D, the *Bgt* microcolony index (MI%) declined to 32% for the BSMV-*TaHDT701as* plants, compared with 54% for the BSMV-γ plants. We then employed the transient gene expression assays to overexpress *TaHDT701* in the wheat epidermal cell and analyzed the haustorial formation of *Bgt*. As shown in Figure 1E, the *Bgt* haustorium index (HI%) increased to 69% on *TaHDT701*-overexpressed (OE-*TaHDT701*) wheat cells, compared with 37% for the empty vector (OE-*EV*) controls, indicating that overexpression of *TaHDT701* remarkably enhances wheat susceptibility to powdery mildew. Together, our results suggest that *TaHDT701* negatively regulates wheat defense responses to powdery mildew.

### 2.2. Wheat TaHDT701 Associates with TaHDA6 and TaHOS15 in The Histone Deacetylase Complex

**Figure 2:**
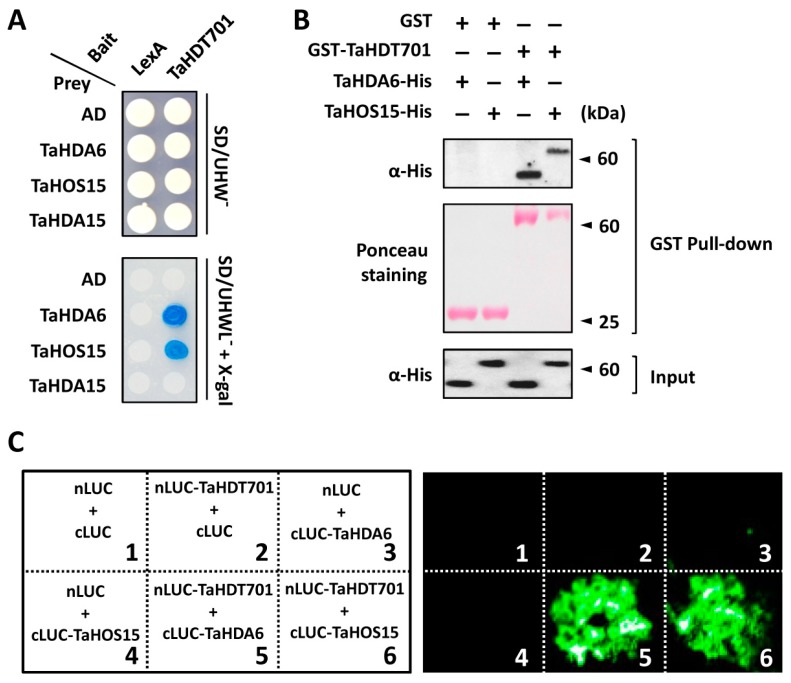
TaHDT701 interacts with TaHDA6 and TaHOS15. (**A**) Analysis of TaHDT701, TaHDA6, and TaHOS15 interaction in yeast-two hybrid. (**B**) In vitro glutathione S-transferase (GST) pull-down analysis of TaHDT701, TaHDA6, and TaHOS15 interaction. TaHDA6-His or TaHOS15-His was incubated with GST or GST-TaHDT701, and the GST resin-bound proteins were immunoblotted with the α-His antibody. (**C**) Luciferase complementation imaging (LCI) analysis of TaHDT701, TaHDA6, and TaHOS15 interaction. N- or C-terminal fragment of luciferase (LUC) (nLUC or cLUC) was fused with indicated proteins and indicated pairs were coexpressed in *N. benthamiana* through agro-infiltration.

It was previously revealed that both of the RPD3 type histone deacetylase AtHDA6 and WD40 repeat protein AtHOS15 could interact with the HD2 type histone deacetylases, including AtHD2A, AtHD2B, and AtHD2C, in Arabidopsis [21,31,32]. To test whether the wheat HD2 type histone deacetylase TaHDT701 could interact with TaHDA6 and TaHOS15, we first performed the yeast two-hybrid (Y2H) assay. Considering that TaHDT701-A, TaHDT701-B, and TaHDT701-D protein shared above 97% sequence identity, we chose TaHDT701-A as a representative of TaHDT701 in the following experiments. The Y2H assay showed that TaHDT701 specifically interacts with both TaHDA6 and TaHOS15, but not with the histone deacetylase TaHDA15 (Figure 2A). We then conducted the glutathione S-transferase (GST) pull-down assay and found that GST-TaHDT701, but not GST alone, pulled down both TaHDA6-His and TaHOS15-His, indicating that TaHDT701 directly interacts with both TaHDA6 and TaHOS15 in vitro (Figure 2B). To further verify the interaction of TaHDT701 with TaHDA6 and TaHOS15 in the plant cell, we performed the luciferase complementation imaging (LCI) assay. As shown in Figure 2C, coexpression of TaHDT701 fused with the N-terminal part of luciferase (nLUC-TaHDT701) and TaHDA6 or TaHOS15 fused with the C-terminal part of luciferase (cLUC-TaHDA6 or cLUC-TaHOS15) generated strong luminescence signals in the leaves of *Nicotiana benthamiana*, which were not detected in the control pairs. These data strongly confirmed that TaHDT701 could interact with both TaHDA6 and TaHOS15 in plant cells.

**Figure 3:**
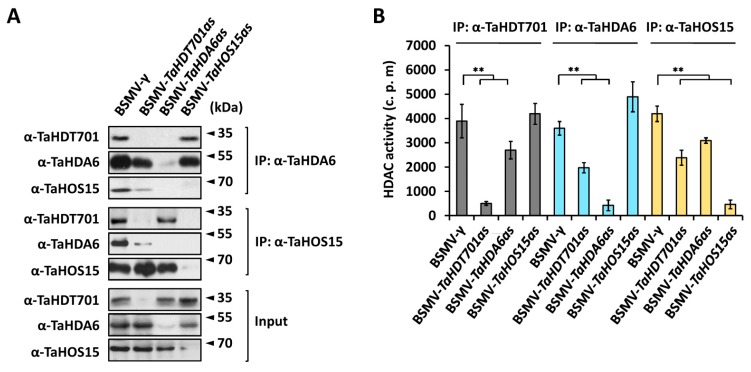
TaHDT701 associates with TaHDA6 and TaHOS15 in a histone deacetylase complex in bread wheat. (**A**) Nuclear co-immunoprecipitation (co-IP) interaction analysis among TaHDT701, TaHDA6, and TaHOS15. Nuclear protein was extracted from the indicated BSMV-VIGS wheat leaves and subjected to immunoprecipitation with the α-TaHDA6 and α-TaHOS15 antibodies. The co-immunoprecipitation of TaHDT701, TaHDA6, and TaHOS15 was probed with the antibodies α-TaHDT701, α-TaHDA6, and α-TaHOS15. (**B**) Immunoprecipitation analysis of endogenous histone deacetylase activity in different background. Nuclear extracts were prepared from the indicated BSMV-VIGS leaves and immunoprecipitated with antibodies α-TaHDT701, α-TaHDA6, and α-TaHOS15. The immunoprecipitated complexes were examined for histone deacetylase activity, which was given as radioactivity (c.p.m.) of [^3^H]-acetate released from an acetylated histone H4 peptide. Data are means from three independent biological repeats. Significant differences were determined using Student’s t-test: ** *p* < 0.01.

To determine the potential interaction among TaHDT701, TaHDA6, and TaHOS15 in bread wheat, we first performed the nuclear co-immunoprecipitation (co-IP) assay in the wheat cultivar Yannong 999 using antibodies specifically targeted to TaHDT701, TaHDA6, and TaHOS15 (Figure S3) [29]. As shown in Figure 3A, TaHDT701, TaHDA6, and TaHOS15 co-immunoprecipitated with each other in the nuclear extract of BSMV-γ plants, which was not detected in the corresponding *TaHDT701-*, *TaHDA6-*, or *TaHOS15*-silenced plants, suggesting that TaHDT701, TaHDA6, and TaHOS15 associate with each other and probably co-exist in the same protein complexes in the nuclei of bread wheat. Interestingly, the association of TaHDA6 with TaHOS15 was markedly weakened in the *TaHDT701*-knockdown plants compared with the control, suggesting that TaHDT701 stabilizes the TaHDA6-TaHOS15 association in bread wheat (Figure 3A). To test whether the TaHDT701-TaHDA6-TaHOS15 complex is associated with histone deacetylase activity in bread wheat, we precipitated endogenous TaHDT701, TaHDA6, and TaHOS15 from wheat cultivar Yannong 999 using specific antibodies and analyzed the deacetylase activity (Figure 3B). Control immunoprecipitations in the corresponding BSMV-VIGS plants showed the background activity (Figure 3B, lane 2,7,12). As shown in Figure 3B, antibodies against TaHDT701, TaHDA6, and TaHOS15 all precipitated with a high level of histone deacetylase activity, which was significantly weakened by knockdown of *TaHDT701*, *TaHDA6*, or *TaHOS15*, indicating that TaHDT701, TaHDA6, and TaHOS15 reside in the same histone deacetylase complex.

### 2.3. Knock-Down of TaHDT701, TaHDA6, and TaHOS15 Compromised Wheat Susceptibility to Powdery Mildew

**Figure 4:**
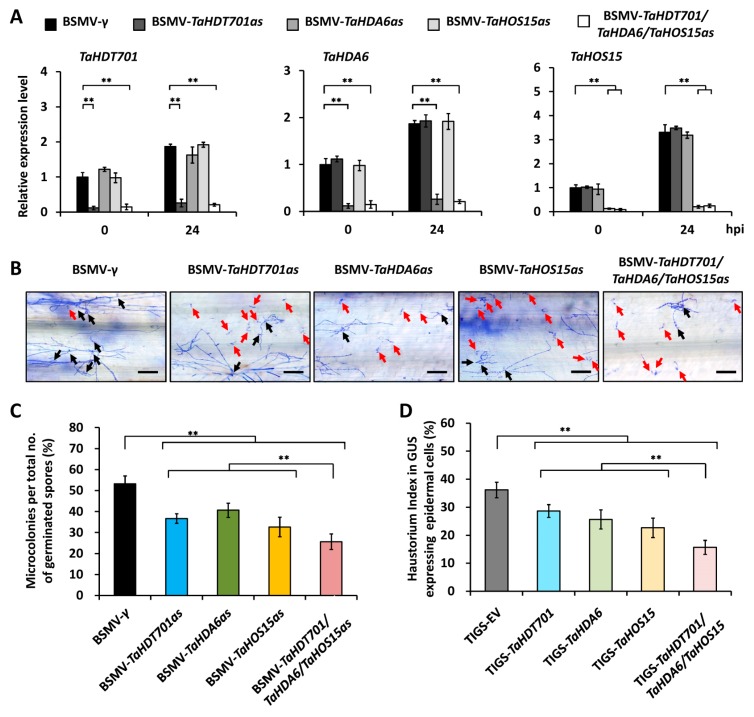
Silencing of *TaHDT701*, *TaHDA6*, and *TaHOS15* compromises wheat susceptibility to *Bgt.* (**A**) Relative transcript abundance of *TaHDT701, TaHDA6,* and *TaHOS15* on wheat plants with different background. The expression level in BSMV-*γ* wheat leave at 0 hpi was set to 1. (**B**) *Bgt* microcolony formation type on wheat plants with different background. Conidia that germinated and finally established microcolonies are indicated with black arrows, and conidia that germinated but failed to establish microcolonies are indicated with red arrows. Bars = 100 μm. (**C**) Statistical analysis of *Bgt* microcolony formation on wheat plants with different background. For each treatment, at least 500 *Bgt-*wheat interaction sites were separately counted. (**D**) Statistical analysis of *Bgt* haustorial formation in wheat epidermal cells with different background. At least 100 cells were analyzed in one experiment. For (**A**), (**C**), and (**D**), three independent biological replicates per treatment were statistically analyzed (t-test, ** *p* < 0.01).

To determine the biological significance of TaHDT701-TaHDA6-TaHOS15 interaction, we silenced endogenous *TaHDT701*, *TaHDA6*, and *TaHOS15* in wheat cultivar Yannong 999 using BSMV-VIGS and analyzed the wheat-powdery mildew interaction. qRT-PCR showed that the expression of *TaHDT701*, *TaHDA6*, and *TaHOS15* was significantly reduced in the wheat leaves infected with BSMV-*TaHDT701as*, BSMV-*TaHDA6as*, and BSMV-*TaHOS15as*, respectively (Figure 4A). As shown in Figure 4B,C, the MI% decreased to 32%, 34%, and 27% in the BSMV-*TaHDT701as*, BSMV-*TaHDA6as*, and BSMV-*TaHOS15as* plants from 53% in the BSMV-γ control plants. Importantly, simultaneous silencing of *TaHDT701*, *TaHDA6*, and *TaHOS15* led to a further reduction of MI%, compared to single silencing of *TaHDT701*, *TaHDA6*, or *TaHOS15* (Figure 4C). Consistently, the HI% decreased by 14%, 18%, and 17% in the *TaHDT701-*, *TaHDA6-*, or *TaHOS15-*silenced wheat cell, which was further reduced by simultaneous silencing of *TaHDT701*, *TaHDA6*, and *TaHOS15* in the single-cell transient gene-silencing assay (Figure 4D). These results strongly suggested that TaHDT701, TaHDA6, and TaHOS15 cooperate to suppress the wheat defense responses to powdery mildew pathogen *Bgt*.

### 2.4. Down-Regulating TaHDT701 Impair The TaHDA6 Occupancy at Chromatin of Defense-Related Genes

**Figure 5:**
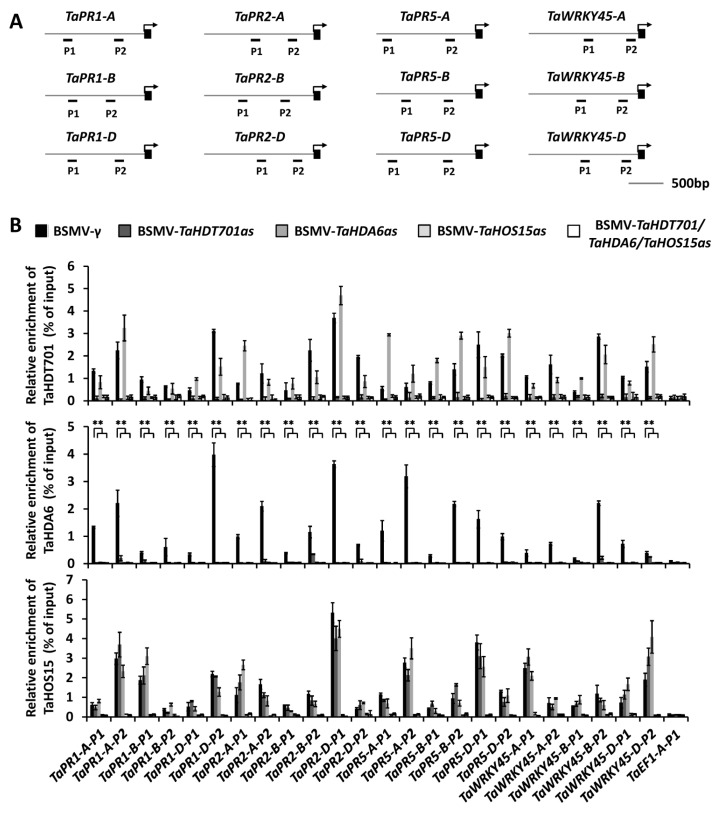
Distribution of TaHDT701, TaHDA6, and TaHOS15 at chromatins of *TaPR1*, *TaPR2*, *TaPR5*, and *TaWRKY45* in different backgrounds. (**A**) Schematic diagram of *TaPR1*, *TaPR2*, *TaPR5*, and *TaWRKY45* genes. Fragments for chromatin immunoprecipitation (ChIP)-qPCR analysis were labeled with numbers. (**B**) The binding of TaHDT701 (upper panel), TaHDA6 (middle panel), and TaHOS15 (lower panel) on the promoter of *TaPR1*, *TaPR2*, *TaPR5*, and *TaWRKY45* in wheat protoplasts analyzed by ChIP-qPCR on wheat plants with different background. The antibodies used for immunoprecipitation are indicated on each graph. The fragments employed for ChIP-qPCR analysis are indicated in Figure 5A. The data are means (±SE) from three independent biological repeats and were analyzed using Student’s t-test: ** *p* < 0.01.

Recent studies revealed that wheat defense-related genes including *TaPR1*, *TaPR2*, *TaPR5*, and *TaWRKY45* are the direct targets of TaHDA6 and TaHOS15 [29]. Based on our finding that TaHDT701, TaHDA6, and TaHOS15 reside in the same histone deacetylase complex, we postulate that TaHDT701 might also associates with chromatin at *TaPR1*, *TaPR2*, *TaPR5*, and *TaWRKY45.* To test this, we conducted a chromatin immunoprecipitation coupled with quantitative PCR (ChIP-qPCR) assay in the wheat cultivar Yannong 999 to analyze the occupancy of these defense-related genes by TaHDT701, TaHDA6, and TaHOS15 (Figure 5A). As shown in Figure 5B, TaHDT701, TaHDA6, and TaHOS15 all accumulate at the promoter regions of *TaPR1*, *TaPR2*, *TaPR5*, and *TaWRKY45* in similar patterns, suggesting that they bind to these promoter regions as a complex. The levels of TaHDT701 and TaHDA6 at the promoter regions of *TaPR1*, *TaPR2*, *TaPR5*, and *TaWRKY45* were remarkably reduced in the BSMV-TaHOS15as infected wheat leaves compared with the BSMV-γ plants, suggesting that TaHOS15 functions as an adaptor to recruit both TaHDA6 and TaHDT701 to these promoters by physical interaction (Figure 5B). Significantly, the level of TaHDA6 at the *TaPR1*, *TaPR2*, *TaPR5*, and *TaWRKY45* promoters was also reduced by knockdown of *TaHDT701*, indicating that the full occupancy of TaHDA6 at promoters of *TaPR1*, *TaPR2*, *TaPR5*, and *TaWRKY45* relies on the presence of TaHDT701, which is consistent with our finding that TaHDT701 stabilizes the TaHDA6-TaHOS15 association (Figure 5B). Taken together, these data indicate that the TaHDT701-TaHDA6-TaHOS15 histone deacetylase complex directly binds to chromatin at defense-related genes including *TaPR1*, *TaPR2*, *TaPR5*, and *TaWRKY45.*

### 2.5. Silencing of TaHDT701, TaHDA6, and TaHOS15 Altered Chromatin State and Transcription Level of Defense-Related Genes under Bgt Infection

**Figure 6:**
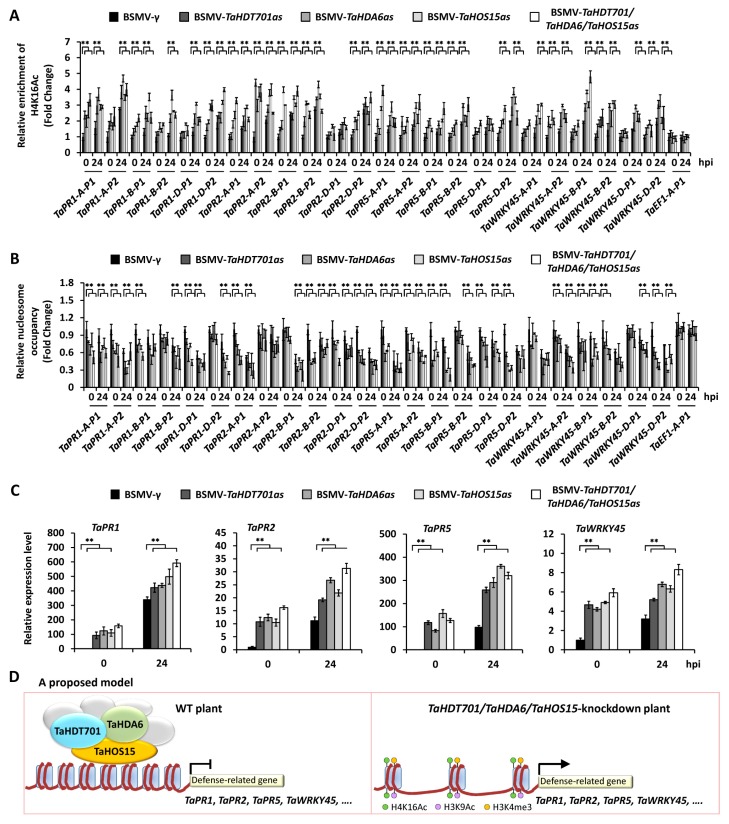
Silencing of *TaHDT701*, *TaHDA6*, and *TaHOS15* affects the histone H4K16Ac, nucleosome distribution and expression levels of *TaPR1*, *TaPR2*, *TaPR5*, and *TaWRKY45*. (**A**) ChIP-qPCR analysis of H4K16Ac at the promoter regions of *TaPR1*, *TaPR2*, *TaPR5*, and *TaWRKY45* on wheat plants with different background. Antibodies α-H4K16Ac were used for immunoprecipitation. Before ChIP-qPCR analysis, the wheat leaves with a typical BMSV symptom were inoculated with *Bgt* conidia for 0 and 24 h. The histone acetylation level in BSMV-*γ* wheat leaves was set to 1.0 at 0 hpi after normalization by histone H4 ChIP. The fragments employed for ChIP-qPCR analysis are indicated in Figure 5A. (**B**) Micrococcal nuclease (MNase) analysis of nucleosome occupancy at promoter regions of *TaPR1*, *TaPR2*, *TaPR5*, and *TaWRKY45* on wheat plants with different background. The nucleosome occupancy levels in BSMV-*γ* wheat leaves at 0 hpi were set to 1.0. (**C**) RT-PCR analysis of *TaPR1*, *TaPR2*, *TaPR5* and *TaWRKY45* expression levels on wheat plants with different background. The expression levels in BSMV-*γ* wheat leaves at 0 hpi were set to 1.0. Three independent biological replicates per treatment were statistically analyzed (t-test, ** *p* < 0.01). For (**A**), (**B**) and (**C**), three independent biological replicates per treatment were statistically analyzed (t-test, ** *p* < 0.01). (**D**) A proposed model of the action of the TaHDT701-TaHDA6-TaHOS15 histone deacetylase complex in regulating wheat defense responses to *Bgt.* As shown in the left panel, the TaHDT701-TaHDA6-TaHOS15 histone deacetylase complex mediates histone deacetylation at the wheat defense-related genes such as *TaPR1*, *TaPR2*, *TaPR5* and *TaWRKY45*, which leads to the suppression of defense-related transcription and defense responses to *Bgt*. In the absence of the TaHDT701-TaHDA6-TaHOS15 histone deacetylase complex (shown in the right panel), chromatin at the defense-related genes is marked by the increased H4K16Ac, H3K9Ac, and H3K4me3, as well as the reduced nucleosome occupancy, thereby stimulating the defense-related transcription and defense responses to *Bgt*.

To examine whether the TaHDT701-TaHDA6-TaHOS15 histone deacetylase complex regulates the histone acetylation at defense-related genes, we silenced endogenous *TaHDT701*, *TaHDA6*, and *TaHOS15* using BSMV-VIGS and analyzed the histone acetylation levels at promoters of *TaPR1*, *TaPR2*, *TaPR5*, and *TaWRKY45* using antibodies specific for acetylated histones H4K16 and H3K9. As shown in Figure 6A and Supplementary Figure S4, the levels of H4K16Ac and H3K9Ac were obviously elevated at the promoter regions of *TaPR1*, *TaPR2*, *TaPR5*, and *TaWRKY45* in the wheat leaves infected with BSMV-*TaHDT701as*, BSMV-*TaHDA6as*, and BSMV-*TaHOS15as* compared with the BSMV-*γ* plants, indicating that TaHDT701, resembling TaHDA6 and TaHOS15, is required for the maintenance of low histone acetylation levels at defense-related genes. Significantly, simultaneous silencing of *TaHDT701*, *TaHDA6*, and *TaHOS15* led to a higher level of H4K16Ac at *TaPR1*, *TaPR2*, *TaPR5*, and *TaWRKY45* promoters, compared to single silencing of *TaHDT701*, *TaHDA6*, and *TaHOS15* (Figure 6A), suggesting that TaHDT701, TaHDA6, and TaHOS15 cooperate in suppressing histone acetylation at defense-related genes.

To determine whether TaHDT701, TaHDA6, and TaHOS15 can affect the permissive histone modifications H3K4me3 and nucleosome distribution at defense-related genes, the levels of histone H3K4Me3 and nucleosome occupancy were analyzed in *TaHDT701*, *TaHDA6*, and *TaHOS15*-knockdown plants. As shown in Supplementary Figure S5, increased abundance of H3K4me3 was observed at the promoter regions of *TaPR1*, *TaPR2*, *TaPR5*, and *TaWRKY45* in the wheat leaves infected with BSMV-*TaHDT701as*, BSMV-*TaHDA6as*, and BSMV-*TaHOS15as* compared with the BSMV-*γ* plants. Subsequent MNase digestion assays showed reduced nucleosome occupancy at the same chromatin regions in the *TaHDT701*, *TaHDA6*, and *TaHOS15*-knockdown plants (Figure 6B). Consistently, the elevated expression of *TaPR1*, *TaPR2*, *TaPR5*, and *TaWRKY45*, but not *TaEF1*, was detected in the BSMV-*TaHDT701as*, BSMV-*TaHDA6as*, and BSMV-*TaHOS15as* plants compared with the BSMV-*γ* plants (Figure 6C, Figure S6). These data revealed that the higher expression of *TaPR1*, *TaPR2*, *TaPR5*, and *TaWRKY45* in the *TaHDT701-*, *TaHDA6-*, and *TaHOS15-*silenced plants was associated with the permissive chromatin state.

## 3. Discussion

In this study, we characterized the biological function of HD2 type histone deacetylase TaHDT701 in the regulation of wheat powdery mildew resistance. The previous study showed that overexpression of *OsHDT701* enhances rice susceptibility to the biotrophic pathogen *M*. *oryzae* and hemibiotrophic pathogen *Xoo*, whereas *OsHDT701*-knockdown leads to increased expression of defense-related genes as well as enhanced resistance to both *M. oryzae* and *Xoo* in rice, indicating that OsHDT701 is a negative regulator of basal defense in rice [18]. Here, we found that *TaHDT701* expression was enhanced by the infection of virulent *Bgt* isolate in bread wheat (Figure 1A). Knockdown of *TaHDT701* expression using virus-induced gene-silencing attenuated wheat susceptibility to the obligate biotrophic pathogen *Bgt,* but overexpression of *TaHDT701* enhanced wheat susceptibility to *Bgt,* suggesting that *TaHDT701,* resembling *OsHDT701*, functions as a negative regulator of plant defense responses to biotrophic pathogens (Figure 1B–E).

Sequence alignment revealed that TaHDT701 shares more than 58% of amino acid sequence identities with AtHD2C from Arabidopsis and has the main structural features of plant-specific HD2 proteins, which include a highly conserved N-terminal octapeptide highlighting the MEFWG sequence, a catalytic domain followed by an extended acidic regulatory domain, and a C-terminal zinc-finger sequence (Figure S2) [33]. Previous studies revealed that the Arabidopsis AtHD2C could interact with RPD3 type deacetylase AtHDA6 and the WD40-repeat protein AtHOS15 [21,31,32]. In addition, these histone deacetylases and WD40-repeat proteins are revealed to get involved in regulating plant abiotic stress responses in Arabidopsis, suggesting that these proteins might function in the same protein complexes [21,31,32]. In this study, Y2H, in vitro pull-down, and LCI assays all indicated that the wheat HD2 type histone deacetylase TaHDT701 directly interacts with the RPD3 type deacetylase TaHDA6 and the WD40-repeat protein TaHOS15 (Figure 2A–C). Through employing the nuclear co-IP and immunoprecipitation of histone deacetylase activity assays, we demonstrated that TaHDA6, TaHDT701, and TaHOS15 reside in the same histone deacetylase complex in the nuclei of bread wheat, suggesting that the RPD3-HD2-WD40 histone deacetylase complexes might be conserved among monocot and dicot plants (Figure 3A,B).

A previous study described the wheat RPD3 type deacetylase TaHDA6 and WD40-repeat protein TaHOS15 as the negative regulators of wheat defense response to *Bgt* [18]. Here, we showed that the simultaneous knockdown of *TaHDT701*, *TaHDA6*, and *TaHOS15* in bread wheat led to the enhanced powdery mildew resistance compared with the single silencing of *TaHDT701*, *TaHDA6*, or *TaHOS15*, suggesting that TaHDT701, TaHDA6, and TaHOS15 indeed cooperate in negatively regulating wheat defense response to *Bgt* (Figure 4). Therefore, our study points to the first example of RPD3-HD2-WD40 histone deacetylase complexes in regulating plant defense responses.

Since RPD3 type histone deacetylases do not directly interact with histones, Arabidopsis WD40 proteins and HD2 type histone deacetylases were proposed to act as scaffold proteins and facilitate histone deacetylation by recruiting RPD3 type histone deacetylases [32,33]. In this study, we demonstrated that the occupancies of TaHDA6 at promoter regions of defense-related genes *TaPR1*, *TaPR2*, *TaPR5*, and *TaWRKY45* were greatly attenuated by knockdown of *TaHOS15* or *TaHDT701* (Figure 5A,B), suggesting that TaHOS15 and TaHDT701 indeed facilitate the recruitment of TaHDA6 to chromatin at defense-related genes. Interestingly, co-IP assay revealed that the interaction between TaHDA6 and TaHOS15 was markedly weakened in the *TaHDT701*-knockdown plants compared with the control, suggesting that TaHDT701 stabilizes the TaHDA6-TaHOS15 association in bread wheat. In addition, Arabidopsis AtHOS15 was revealed to function as a DCAF (CUL4- and DDB1-associated factors) protein and could mediate the ubiquitination and degradation of histone deacetylase AtHD2C [22,32]. Here, our study revealed that TaHOS15 associates with TaHDT701 and TaHDA6 in crop plants, which opens up other areas for further research such as characterizing the potential role of TaHOS15 in regulating degradation of these histone deacetylases.

Modulation of gene expression through crosstalk between histone acetylation, methylation, and nucleosome occupancy has been reported previously [21,34]. For instance, Arabidopsis histone deacetylase AtHDA6 can interact with histone demethylase FLD and histone methyltransferases SUVH4, SUVH5, and SUVH6 to regulate both histone acetylation and methylation [21,35]. In addition, chromatin at defense-related genes in Arabidopsis CHROMATIN ASSEMBLY FACTOR 1 (CAF-1) mutants is marked by low nucleosome occupancy and high H3K4me3 [36]. In this study, increased histone acetylation at promoters of *TaPR1*, *TaPR2*, *TaPR5*, and *TaWRKY45* was found in *TaHDT701*, *TaHDA6*, and *TaHOS15-*knockdown plants (Figure 6A). In addition, simultaneous silencing of *TaHDT701*, *TaHDA6*, and *TaHOS15* had enhanced histone acetylation at promoters of *TaPR1*, *TaPR2*, *TaPR5*, and *TaWRKY45* compared with single silencing of *TaHDT701*, *TaHDA6*, and *TaHOS15* (Figure 6A), suggesting that TaHDT701, TaHDA6, and TaHOS15 cooperate to suppress histone acetylation at defense-related promoters. At the same time, increased H3K4Me3 and reduced nucleosome occupancy were observed at promoters of *TaPR1*, *TaPR2*, *TaPR5*, and *TaWRKY45* (Figure 6B and Figure S4), suggesting that altered histone modifications and nucleosome occupancy are closely linked during defense gene priming in *TaHDT701*, *TaHDA6*, and *TaHOS15-*knockdown plants, whose underlying mechanism remains to be uncovered in future research.

These results allowed us to establish a model about how the TaHDT701-TaHDA6-TaHOS15 histone deacetylase complex negatively regulates the defense responses to powdery mildew in bread wheat. As shown in Figure 6D, the TaHDT701-TaHDA6-TaHOS15 histone deacetylase complex binds to chromatin at defense-related genes including *TaPR1*, *TaPR2*, *TaPR5*, and *TaWRKY45*, where they catalyze the histone deacetylation in chromatin to suppress the expression of these defense-related genes. In the absence of the TaHDT701-TaHDA6-TaHOS15 histone deacetylase complex, chromatin at the defense-related genes resides in a primed gene expression state marked by the increased H4K16Ac, H3K9Ac, and H3K4me3, as well as the reduced nucleosome occupancy, thereby stimulating the defense-related transcription and defense responses to *Bgt*.

## 4. Materials and Methods

### 4.1. Plant and Fungal Materials and Treatments

The bread wheat (*Triticum aestivum* L.) cultivar Yannong 999, which is susceptible to *Bgt* (*Blumeria graminis* f. sp. *tritici*) strain E09, was employed in this study. The bread wheat and *Nicotiana benthamiana* were grown in a glasshouse with a 16 h light, 20 °C/8 h dark, 18 °C regime with 70% relative humidity. *Bgt* strain E09 was maintained on wheat cultivar Yannong 999 plants. Wheat plant inoculation with *Bgt* was described [29]. Briefly, the fully emerged wheat leaves were inoculated with *Bgt* conidia by gently shaking conidia from leaves of a *Bgt*-infected wheat plant. The inoculated wheat leaves were incubated in a growth chamber with a 16 h light, 20 °C/8 h dark, 18 °C regime with 70% relative humidity.

### 4.2. Quantitative Reverse-Transcription Polymerase Chain Reaction (qRT-PCR)

Total RNA was isolated from wheat leaves as described previously [29]. The purified RNA samples were reverse-transcribed to cDNA using the Superscript II reverse transcriptase (Invitrogen, Carlsbad, CA, USA) according to the manufacturer’s instructions. The quantative PCR assay was performed as described previously [29]. The expression of *TaGADPH*, *TaHDT701*, *TaHDA6*, *TaHOS15*, *TaPR1*, *TaPR2*, *TaPR5*, and *TaWRKY45* were analyzed using the primers listed in Table S1. Statistical significance was evaluated using Student’s t test.

### 4.3. BSMV-Mediated Gene-Silencing (BSMV-VIGS) Assays

BSMV-mediated gene-silencing (BSMV-VIGS) assays were performed as previously described [29]. Briefly, fragments of ~200 bp of *TaHDT701* were amplified using the primers listed in Table S1 and then cloned into the pCa-γbLIC vector to create the BSMV-*TaHDT701as* construct. The BSMV-*TaHDA6as* and BSMV-*TaHOS15as* constructs were derived from a previous study [29]. The BSMV-mediated gene silencing in wheat leaves was performed as described previously [37]. For simultaneous silencing of *TaHDT701*, *TaHDA6*, and *TaHOS15* (BSMV-*TaHDT701/TaHDA6/TaHOS15as*), the *N. benthamiana* leave saps containing BSMV-*TaHDT701as*, BSMV-*TaHDA6as*, and BSMV-*TaHOS15as* were mixed and co-inoculated onto the wheat leaves. About three weeks after BSMV infection, young fully emerged leaves with BSMV virus symptoms were inoculated with conidia of *Bgt* strain E09. About 72 h post-*Bgt* inoculation, the fungal epiphytic structure was observed using Coomassie blue staining, as described previously [29].

### 4.4. Single-Cell Transient Gene-Silencing and Expression Assays

Fragments of ~200 bp of *TaHDT701* were amplified using the primers listed in Table S1 and then cloned into the pIPKb007 vector to create the TIGS-TaHDT701 construct. The TIGS-TaHDA6, and TIGS-TaHOS15 constructs were derived from a previous study [29]. In addition, the coding region of *TaWRKY76* was amplified using the primers listed in Supplementary Table S1 and then cloned into the pIPKb001 vector. The single-cell transient gene-silencing and expression was conducted essentially as described in [29].

### 4.5. Yeast Two-Hybrid Analysis

For yeast two-hybrid analysis, coding regions of *TaHDT701*, *TaHDA6*, and *TaHOS15* were amplified through employing the primers listed in Table S1 and then ligated into the vectors pLexA and pB42AD. The yeast two-hybrid assays were performed as previously described [38].

### 4.6. GST Pull-Down Assays

For the GST pull-down analysis, coding regions of *TaHDT701,* TaHDA6, and TaHOS15 were amplified using the primers listed in Table S1 and ligated into the vectors pET32a and pGEX4T-1. The yeast two-hybrid assays were performed as previously described [38]. The co-precipitated TaHDA6-His and TaHOS15-His were analyzed using immunoblotting analysis with α-His antibody (CWBIO).

### 4.7. Luciferase Complementation Imaging (LCI) Assays

The Luciferase complementation imaging assays were performed as described by Kong and Chang [38]. Briefly, the coding fragments of *TaHDT701* were amplified through employing the primers listed in Table S1 and ligated into the vectors pCAMBIA-nLUC to express protein fusions to the luciferase N-terminal domain, respectively. *Agrobacterium tumefaciens* strain GV3101 containing CAMBIA-nLUC and pCAMBIA-cLUC derivative constructs were co-infiltrated into *N. benthamiana* leaves. The LCI images of the infiltrated plant leaves were obtained with cooled CCD imaging apparatus (Andor, Belfast, GB) at 48 h post infiltration.

### 4.8. Nuclear Co-Immunoprecipitation (Co-IP) Assays

The nuclear co-immunoprecipitation (co-IP) assays were performed as described previously by Liu et al. [29]. Briefly, young, fully emerged BSMV-VIGS leaves with virus symptoms were detached from the wheat cultivar Yannong 999 plants and ground in liquid nitrogen. Wheat nuclei were isolated in the Honda buffer (2.5% Ficoll 400, 5% dextran T40, 0.4 M sucrose, 25 mM Tris-HCl, pH 7.5, 10 mM MgCl_2_, 1 mM DTT, 1 mM PMSF, and 1× complete protease inhibitor cocktail). The nuclear extracts were digested with DNase I (Sigma-Aldrich, St. Louis., MO, USA) to eliminate the potential DNA-protein interactions during the co-IP. The immunoprecipitation and co-immunoprecipitation of TaHDT701, TaHDA6, and TaHOS15 was analyzed by immunoblotting with α-TaHDT701 (the sequence DTAAPSKSKAAAKDVGKSNKDD was used as an epitope for generating antibody against TaHDT701), α-TaHDA6 [29], and α-TaHOS15 [29].

### 4.9. Immunoprecipitation of Histone Deacetylase Activity

Immunoprecipitation of histone deacetylase activity was performed as described with modification [39]. Briefly, the nuclear protein extracts were prepared from BSMV-VIGS wheat leaves and immunoprecipitated with antibodies α-TaHDT701, α-TaHDA6, and α-TaHOS15, as described above. After being washed with IPH buffer (50 mM Tris-HCl, pH 8.0, 150 mM NaCl, 5 mM EDTA, 1 mM DTT, 0.5% Nonidet P-40, 1 mM phenylmethylsulfonyl fluoride), the antibody complexes were incubated with a synthetic acetylated H4 peptide labeled with tritium [^3^H]. The released [^3^H] acetate was extracted with ethyl acetate and quantified using scintillation counting.

### 4.10. Chromatin Immunoprecipitation (ChIP) Assays

Chromatin immunoprecipitation assays were carried out as described with modification [40]. Briefly, young, fully emerged BSMV-VIGS leaves with virus symptoms were inoculated with conidia of *Bgt* strain E09. After the indicated time, wheat leaves were fixed in 1% (v/v) formaldehyde and the nuclei were isolated and lysed. Cross-linked chromatin was sonicated and precleared with 50 µL of Dynabeads protein A (Invitrogen, Carlsbad, CA, USA) and then immunoprecipitated using antibodies α-TaHDT701, α-TaHDA6, α-TaHOS15, α-histone H4 (Millipore, Billerica, MA, USA), α-H4K16Ac (Millipore, Billerica, MA, USA), α-H3K9Ac (Millipore, Billerica, MA, USA), and α-H3K4me3 (Millipore, Billerica, MA, USA). DNA recovery after ChIP was quantified as the percentage of input. Relative enrichment of H4K16Ac, H3K9Ac, and H3K4me3 was calculated after normalizing the histone acetylation and methylation ChIP with histone H4 and H3 ChIP, respectively. Real-time PCRs were performed using gene-specific primers listed in Supplementary Table S1.

### 4.11. Nucleosome Occupancy Micrococcal Nuclease (MNase) Assay

Nucleosome occupancy micrococcal nuclease (MNase) assays were conducted as described by Shu et al. [41]. Briefly, young, fully emerged BSMV-VIGS leaves with virus symptoms were inoculated with conidia of *Bgt* strain E09. After the indicated time, wheat leaves were cross-linked, and the nuclei were extracted. After digestion using MNase (New England BioLabs, Ipswich, MA, USA), DNA was recovered and analyzed using real-time PCRs using gene-specific primers listed in Supplementary Table S1. Extracted nuclei without MNase treatment were employed as input control.

### 4.12. Accession Numbers

Sequence data from this study can be found in the GenBank database (http://www.ncbi.nlm.nih.gov/) under the following accession numbers: TaHDT701-A: MN295033, TaHDT701-B: MN295034, TaHDT701-D: MN295035, TaHDA6-A: MH556916, TaHDA6-B: MH556917, TaHDA6-D: MH556918, TaHOS15-A: MH556919, TaHOS15-B: MH556920, TaHOS15-D: MH556921, OsHDT701: Os05g51830.

## 5. Conclusions

In this study, we demonstrated that wheat HD2 type histone deacetylase TaHDT701 associated with both the RPD3 type histone deacetylase TaHDA6 and the WD40-repeat protein TaHOS15 to constitute a histone deacetylase complex. In addition, the TaHDT701-TaHDA6-TaHOS15 histone deacetylase complex was shown to negatively regulate the wheat defense responses to *Bgt* by modulating the chromatin state at defense-related genes. Therefore, this study not only enriches our knowledge about the epigenetic regulation of wheat powdery mildew resistance, but also points to the first example of HD2-RPD3-WD40 histone deacetylase complexes in regulating plant defense responses.

## Supplementary Materials

Supplementary materials can be found at https://www.mdpi.com/1422-0067/21/7/2640/s1.

## Abbreviations

*Bgt*	*Blumeria graminis* f.sp. *tritici*
BSMV	barley stripe mosaic virus
BSMV-VIGS	barley stripe mosaic virus-induced gene-silencing
ChIP	chromatin immunoprecipitation
ChIP-qPCR	chromatin immunoprecipitation coupled with quantitative PCR
EF1	elongation factor 1
EV	empty vector
GST	glutathione S-transferase
H3K4Me3	trimethylation of histone H3 lysine 4
H3K9Ac	acetylation of histone H3 lysine 9
H4K16Ac	acetylation of histone H4 lysine 16
HD2	type-II histone deacetylase
HDAC	histone deacetylase
HI%	haustorium index
hpi	hours post-inoculation
LCI	luciferase complementation imaging
MI%	microcolony index
MNase	micrococcal nuclease
qRT-PCR	quantitative reverse transcription-polymerase chain reaction
RPD3	reduced potassium dependency protein 3
TIGS	transient induced gene-silencing
WDR protein	WD40-repeat protein
Y2H	yeast two-hybrid.

## References

[1] Parlange F., Roffler S., Menardo F., Ben-David R., Bourras S., McNally K.E., Oberhaensli S., Stirnweis D., Buchmann G., Wicker T. (2015). Genetic and molecular characterization of a locus involved in avirulence of *Blumeria graminis* f. sp. *tritici* on wheat *Pm3* resistance alleles. Fungal Genet. Boil..

[2] Zhu X., Yang K., Wei X., Zhang Q., Rong W., Du L., Ye X., Qi L., Zhang Z. (2015). The wheat AGC kinase TaAGC1 is a positive contributor to host resistance to the necrotrophic pathogen *Rhizoctonia cerealis*. J. Exp. Bot..

[3] Zou S., Wang H., Li Y., Kong Z., Tang D. (2017). The NB-LRR gene *Pm60* confers powdery mildew resistance in wheat. New Phytol..

[4] Geng S., Kong X., Song G., Jia M., Guan J., Wang F., Qin Z., Wu L., Lan X., Li A. (2018). DNA methylation dynamics during the interaction of wheat progenitor Aegilops tauschii with the obligate biotrophic fungus *Blumeria graminis* f. sp. *tritici*. New Phytol..

[5] He H., Zhu S., Zhao R., Jiang Z., Ji Y., Ji J., Qiu D., Li H.-J., Bie T. (2018). *Pm21*, encoding a typical CC-NBS-LRR protein, confers broad-spectrum resistance to wheat powdery mildew disease. Mol. Plant.

[6] Koller T., Brunner S., Herren G., Hurni S., Keller B. (2018). Pyramiding of transgenic *Pm3* alleles in wheat results in improved powdery mildew resistance in the field. Theor. Appl. Genet..

[7] Xing L., Hu P., Liu J., Witek K., Zhou S., Xu J., Zhou W., Gao L., Huang Z., Zhang R. (2018). *Pm21* from *Haynaldia villosa* encodes a CC-NBS-LRR protein conferring powdery mildew resistance in wheat. Mol. Plant.

[8] Zhang Y., Bai Y., Wu G., Zou S., Chen Y., Gao C., Tang D. (2017). Simultaneous modification of three homoeologs of *TaEDR1* by genome editing enhances powdery mildew resistance in wheat. Plant J..

[9] Adachi H., Tsuda K. (2019). Convergence of cell-surface and intracellular immune receptor signalling. New Phytol..

[10] Buscaill P., Rivas S. (2014). Transcriptional control of plant defence responses. Curr. Opin. Plant Boil..

[11] Jones A.M., Vance R.E., Dangl J.L. (2016). Intracellular innate immune surveillance devices in plants and animals. Science.

[12] Ramirez-Prado J.S., Piquerez S.J.M., Bendahmane A., Hirt H., Raynaud C., Benhamed M. (2018). Modify the histone to win the battle: Chromatin dynamics in plant–pathogen interactions. Front. Plant Sci..

[13] Tsuda K., Somssich I. (2015). Transcriptional networks in plant immunity. New Phytol..

[14] Ding B., Wang G.-L. (2015). Chromatin versus pathogens: The function of epigenetics in plant immunity. Front. Plant Sci..

[15] Kong L., Liu Y., Wang X., Chang C. (2020). Insight into the role of epigenetic processes in abiotic and biotic stress response in wheat and barley. Int. J. Mol. Sci..

[16] Espinas N.A., Saze H., Saijo Y. (2016). Epigenetic control of defense signaling and priming in plants. Front. Plant Sci..

[17] Kurdistani S.K., Grunstein M. (2003). Histone acetylation and deacetylation in yeast. Nat. Rev. Mol. Cell Boil..

[18] Ding B., Bellizzi M.D.R., Ning Y., Meyers B.C., Wang G.-L. (2012). HDT701, a histone H4 deacetylase, negatively regulates plant innate immunity by modulating histone H4 acetylation of defense-related genes in rice. Plant Cell.

[19] Pandey R. (2002). Analysis of histone acetyltransferase and histone deacetylase families of *Arabidopsis thaliana* suggests functional diversification of chromatin modification among multicellular eukaryotes. Nucleic Acids Res..

[20] Mehdi S., Derkacheva M., Ramström M., Kralemann L., Bergquist J., Hennig L. (2015). The WD40 domain protein MSI1 functions in a histone deacetylase complex to fine-tune abscisic acid signaling. Plant Cell.

[21] Luo M., Wang Y.-Y., Liu X., Yang S., Lu Q., Cui Y., Wu K. (2012). HD2C interacts with HDA6 and is involved in ABA and salt stress response in *Arabidopsis*. J. Exp. Bot..

[22] Park J., Lim C.J., Shen M., Park H.J., Cha J.-Y., Iniesto E., Rubio V., Mengiste T., Zhu J.-K., Bressan R.A. (2018). Epigenetic switch from repressive to permissive chromatin in response to cold stress. Proc. Natl. Acad. Sci. USA.

[23] Choi S.-M., Song H.-R., Han S.-K., Han M., Kim C.-Y., Park J., Lee Y., Jeon J.-S., Noh Y.-S., Noh B. (2012). HDA19 is required for the repression of salicylic acid biosynthesis and salicylic acid-mediated defense responses in *Arabidopsis*. Plant J..

[24] Wang Y., Hu Q., Wu Z., Wang H., Han S., Jin Y., Zhou J., Zhang Z., Jiang J., Shen Y. (2017). HISTONE DEACETYLASE 6 represses pathogen defense responses in *Arabidopsis thaliana*. Plant Cell Environ..

[25] Wu K., Zhang L., Zhou C., Yu C.-W., Chaikam V. (2008). HDA6 is required for jasmonate response, senescence and flowering in *Arabidopsis*. J. Exp. Bot..

[26] Zhou C., Zhang L., Duan J., Miki B., Wu K. (2005). HISTONE DEACETYLASE19 is involved in jasmonic acid and ethylene signaling of pathogen response in *Arabidopsis*. Plant Cell.

[27] Wang C., Gao F., Wu J., Dai J., Wei C., Li Y. (2010). *Arabidopsis* putative deacetylase AtSRT2 regulates basal defense by suppressing *PAD4*, *EDS5* and *SID2* expression. Plant Cell Physiol..

[28] Latrasse D., Jégu T., Li H., De Zelicourt A., Raynaud C., Legras S., Gust A., Samajova O., Veluchamy A., Rayapuram N. (2017). MAPK-triggered chromatin reprogramming by histone deacetylase in plant innate immunity. Genome Boil..

[29] Liu J., Zhi P., Wang X., Fan Q., Chang C. (2018). Wheat WD40-repeat protein TaHOS15 functions in a histone deacetylase complex to fine-tune defense responses to *Blumeria graminis* f.sp. *tritici*. J. Exp. Bot..

[30] Appels R., Eversole K., Stein N., Feuillet C., Keller B., Rogers J., Pozniak C.J., Choulet F., Distelfeld A., The International Wheat Genome Sequencing Consortium (IWGSC) (2018). Shifting the limits in wheat research and breeding using a fully annotated reference genome. Science.

[31] Luo M., Wang Y.-Y., Liu X., Yang S., Wu K. (2012). HD2 proteins interact with RPD3-type histone deacetylases. Plant Signal. Behav..

[32] Park H.J., Baek D., Cha J.-Y., Liao X., Kang S.-H., McClung C.R., Lee S.Y., Yun D.-J., Kim W.-Y. (2019). HOS15 interacts with the histone deacetylase HDA9 and the evening complex to epigenetically regulate the floral activator GIGANTEA. Plant Cell.

[33] Bourque S., Jeandroz S., Grandperret V., Lehotai N., Aimé S., Soltis D., Miles N., Melkonian M., Deyholos M., Leebens-Mack J. (2016). The evolution of HD2 proteins in green plants. Trends Plant Sci..

[34] Chen L.-T., Luo M., Wang Y.-Y., Wu K. (2010). Involvement of *Arabidopsis* histone deacetylase HDA6 in ABA and salt stress response. J. Exp. Bot..

[35] Yu C.-W., Tai R., Wang S.-C., Yang P., Luo M., Yang S., Cheng K., Wang W.-C., Cheng Y.-S., Wu K. (2017). HISTONE DEACETYLASE6 acts in concert with histone methyltransferases SUVH4, SUVH5, and SUVH6 to regulate transposon silencing. Plant Cell.

[36] Mozgova I., Wildhaber T., Liu Q., Mansour E.A., L’Haridon F., Métraux J.-P., Gruissem W., Hofius D., Hennig L. (2015). Chromatin assembly factor CAF-1 represses priming of plant defence response genes. Nat. Plants.

[37] Yuan C., Li C., Yan L., Jackson A.O., Liu Z., Han C., Yu J., Li D. (2011). A high throughput barley stripe mosaic virus vector for virus induced gene silencing in monocots and dicots. PLoS ONE.

[38] Kong L., Chang C. (2018). Suppression of wheat TaCDK8/TaWIN1 interaction negatively affects germination of *Blumeria graminis* f.sp. *tritici* by interfering with very-long-chain aldehyde biosynthesis. Plant Mol. Biol..

[39] Taunton J., Hassig C.A., Schreiber S.L. (1996). A mammalian histone deacetylase related to the yeast transcriptional regulator Rpd3p. Science.

[40] Ding Y., Ndamukong I., Xu Z., Lapko H., Fromm M., Avramova Z. (2012). ATX1-generated H3K4me3 is required for efficient elongation of transcription, not initiation, at ATX1-regulated genes. PLoS Genet..

[41] Shu H., Gruissem W., Hennig L. (2013). Measuring Arabidopsis chromatin accessibility using DNase I-polymerase chain reaction and DNase I-chip assays. Plant Physiol..

