# Supplementary material for: Histone Deacetylase TaHDT701 Functions in TaHDA6-TaHOS15 Complex to Regulate Wheat Defense Responses to Blumeria graminis f.sp. tritici
Source: Int J Mol Sci. 2020 Apr 10;21(7):2640. doi: 10.3390/ijms21072640 (PMC7178159; doi:10.3390/ijms21072640)
Supplement: Supplementary file 1 [file ijms-21-02640-s001.pdf]

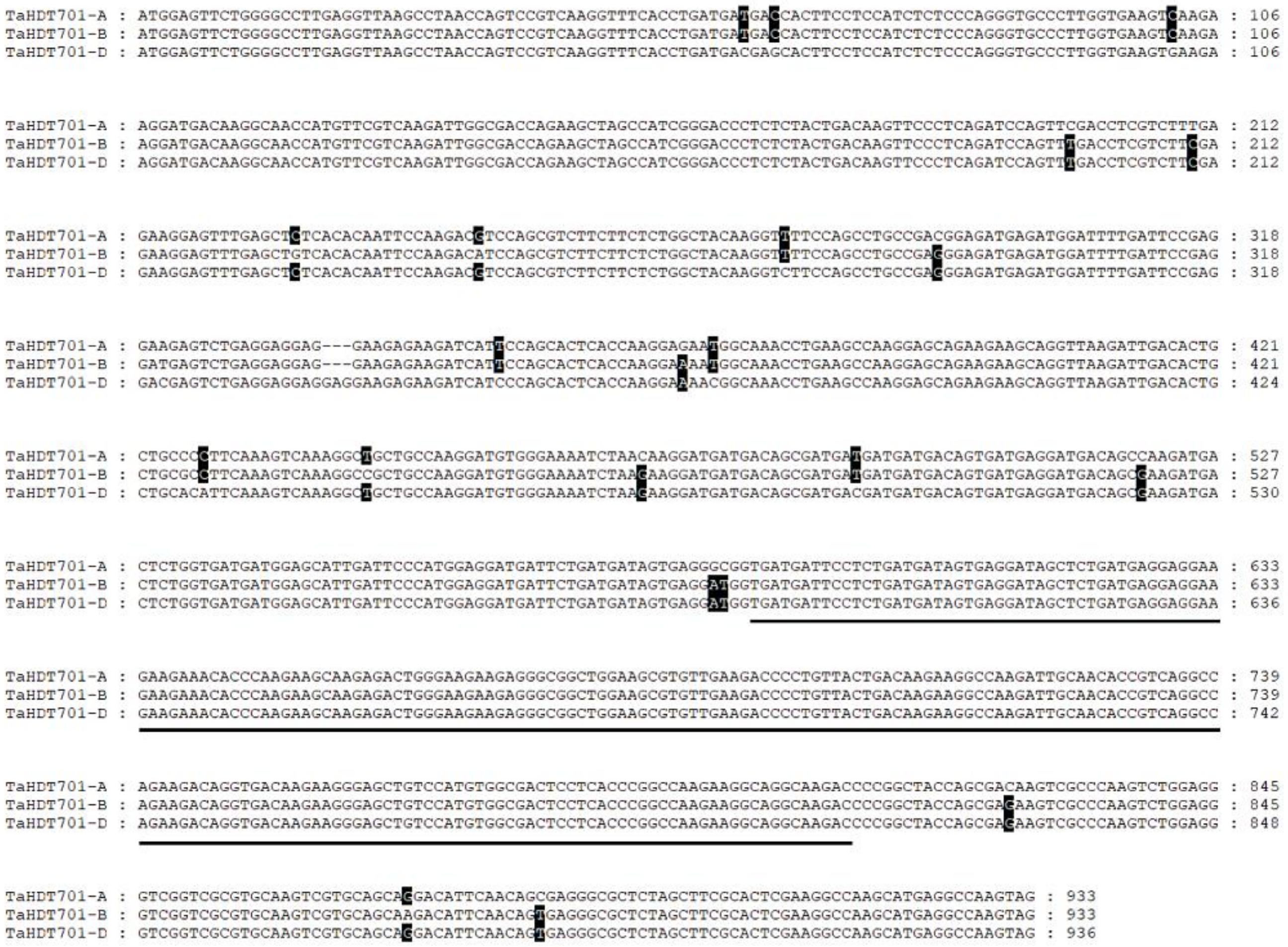

Figure S1. Nucleotide sequences alignment at the coding regions of allelic *TaHDT701-A*, *TaHDT701-B*, and *TaHDT701-D*. The nucleotide sequences of *TaHDT701-A*, *TaHDT701-B*, or *TaHDT701-D* are derived from wheat A, B or D genome, respectively. Variations among *TaHDT701-A*, *TaHDT701-B*, or *TaHDT701-D* nucleotide sequences are shaded in dark. Regions chosen for the *TaHDT701* silencing are underlined in dark.

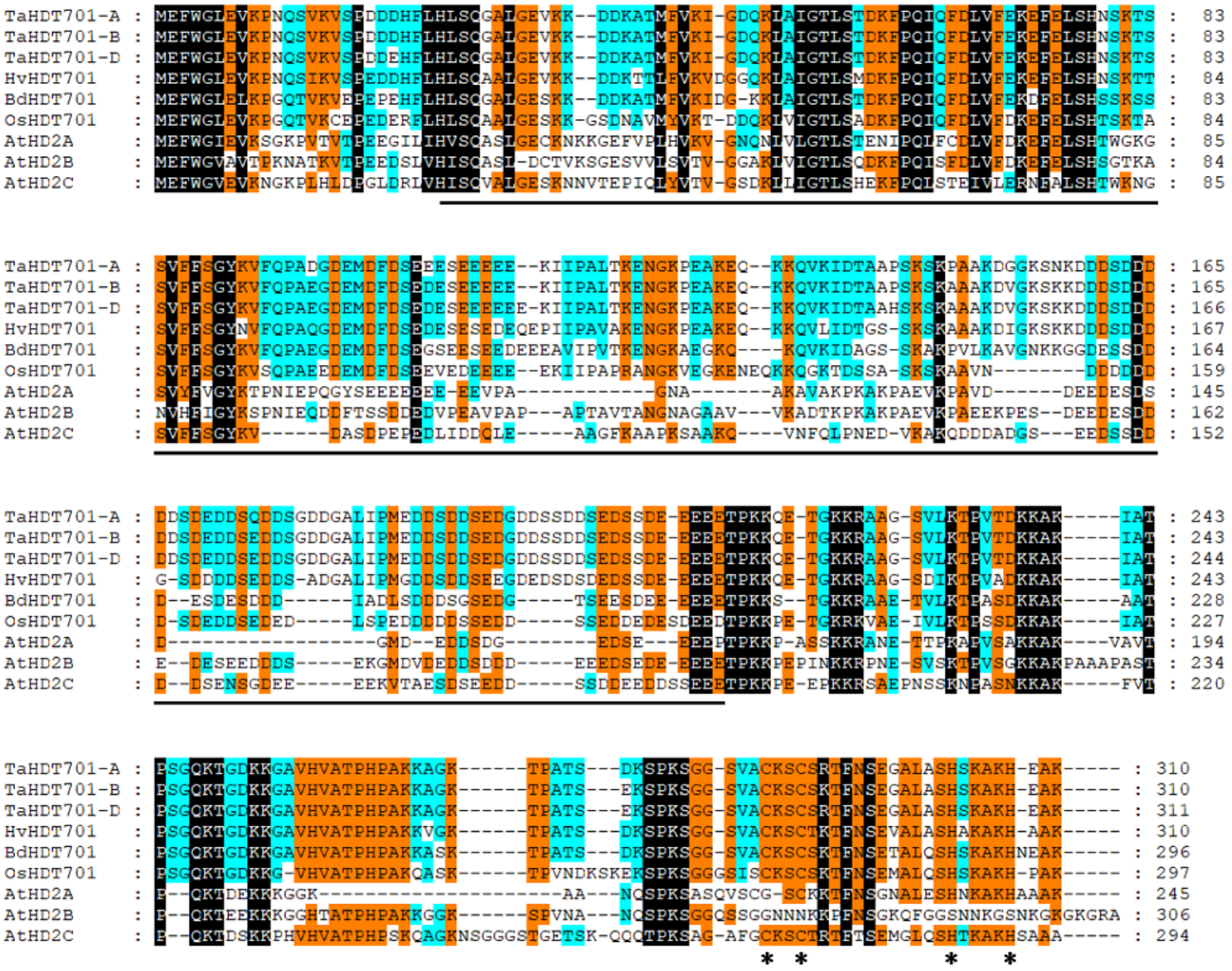

Figure S2. Sequences comparison of plant HD2 proteins. TaHDT701-A, TaHDT701-B, and TaHDT701-D proteins had more than 51% amino acid sequence identities with HvHDT701 (EU348775) from *Hordeum vulgare*, BdHDT701 (XP_003567768) from *Brachypodium distachyon*, OsHDT701 (Os05g51830) from *Oryza sativa* and AtHD2A (AT3G44750), AtHD2B (AT5G22650), and AtHD2C (AT5G03740) from *Arabidopsis thaliana*. Identical residues among 9 protein sequences are shaded in dark. The catalytic domain followed by an extended acidic regulatory domain is underlined. The cysteine and histidine residues of the zinc finger are marked by the dark asterisk.

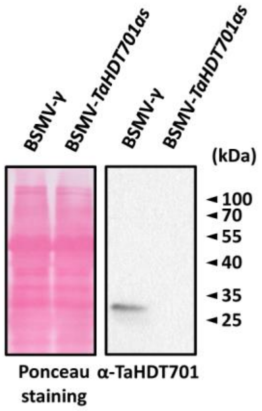

Figure S3. Immunoblot analysis of wheat total protein using the α-TaHDT701 antibody. Total proteins were extracted from the wheat leaves separately infected with BSMV-*γ* and BSMV-*TaHDT701as* and subjected to immunoblotting with the α-TaHDT701 antibody. The same protein gel was stained with Ponceau Red to show equal loading.

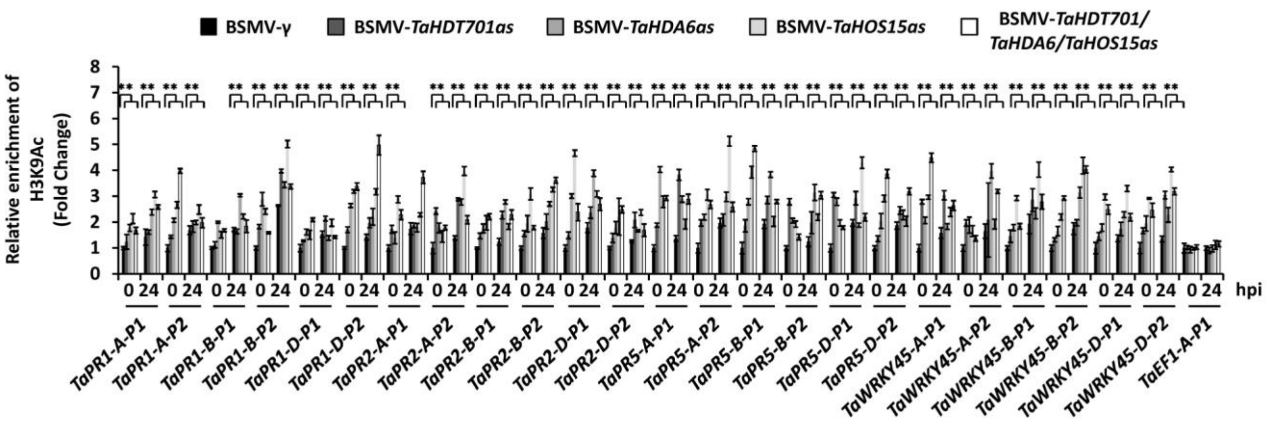

Figure S4. ChIP-qPCR analysis of H3K9Ac at promoter regions of *TaPR1*, *TaPR2*, *TaPR5* and *TaWRKY45* on wheat plants with different background. Antibodies α-H3K9Ac were used for immunoprecipitation. Before ChIP-qPCR analysis, the wheat leaves with a typical BMSV symptom were inoculated with *Bgt* conidia for 0 and 24 hours. The histone acetylation level in BSMV-*γ* wheat leaves were set to 1.0 at 0 hpi after normalization by histone H3 ChIP. The fragments employed for ChIP-qPCR analysis are indicated in Figure 5A.

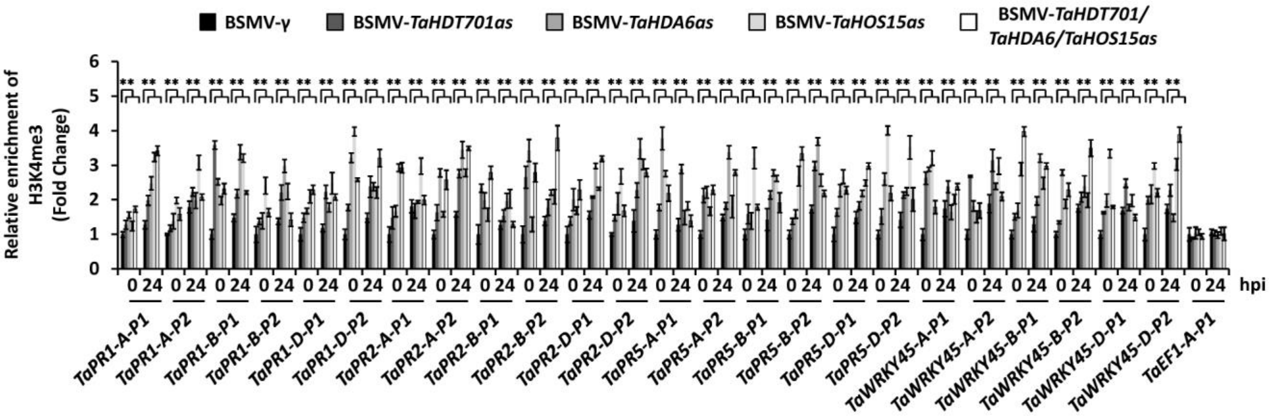

Figure S5. ChIP-qPCR analysis of H3K4me3 at promoter regions of *TaPR1*, *TaPR2*, *TaPR5* and *TaWRKY45* on wheat plants with different background. Antibodies α-H3K4me3 were used for immunoprecipitation. Before ChIP-qPCR analysis, the wheat leaves with a typical BMSV symptom were inoculated with *Bgt* conidia for 0 and 24 hours. The histone acetylation level in BSMV-*γ* wheat leaves were set to 1.0 at 0 hpi after normalization by histone H3 ChIP. The fragments employed for ChIP-qPCR analysis are indicated in Figure 5A.

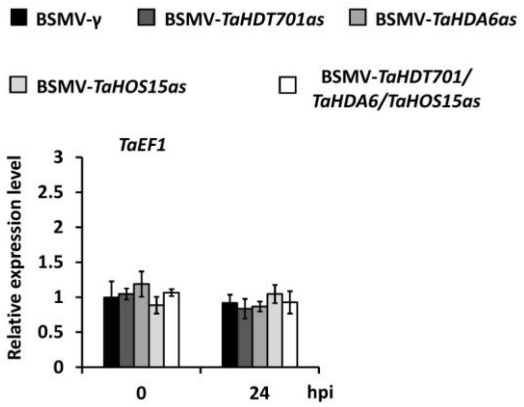

Figure S6. RT-PCR analysis of *TaEF1* expression levels on wheat plants with different background. The expression levels in BSMV-*γ* wheat leaves at 0 hpi were set to 1.0.

Table S1. Primers used in this study

Primer Name	Sequence	Annotation
TaHDT701-F1	5' AAATCCATCCGTCCGTCCGTC3'	First round nested PCR for *TaHDT701* cloning, forward (F) primer
TaHDT701-R1	5' ATAAGGATGAGGCTAGGTAAG3'	First round nested PCR for *TaHDT701* cloning, reverse (R) primer
TaHDT701-F2	5' CCGGTTGCCATGGAGTTCTG3'	Second round nested PCR for *TaHDT701* cloning, F primer
TaHDT701-R2	5' CATAGGAGGCAGCCAGCCAGC3'	Second round nested PCR for *TaHDT701* cloning, R primer
BSMV-*TaHDT701as*-F	5'AAGGAAGTTTAAGATGTCTTGCCTGCCTTCTTGG3'	For construct of BSMV-*TaHDT701as*, F primer
BSMV-*TaHDT701as*-R	5'AACCACCACCACCGTCTTGATGATTCCTCTGATGATAG3'	For construct of BSMV-*TaHDT701as*, R primer
TIGS-TaHDT701-F	5'GGGGACAAGTTTGTACAAAAAAGCAGGCTTCGTCTTGCCTGCCTTCTTGG3'	For construct of TIGS-TaHDT701, F primer
TIGS-TaHDT701-R	5'GGGGACCACTTTGTACAAGAAAGCTGGGTCTGATGATTCCTCTGATGATAG3'	For construct of TIGS-TaHDT701, R primer
pLexA-TaHDT701-F	5'GGGGACAAGTTTGTACAAAAAAGCAGGCTTCATGGAGTTCTGGGGCCTTGAG3'	For construct of pLexA-TaHDT701, F primer
pLexA-TaHDT701-R	5'GGGGACCACTTTGTACAAGAAAGCTGGGTCCTACTTGGCCTCATGCTTGG3'	For construct of pLexA-TaHDT701, R primer
pCAMBIA-YN-TaHDT701-F	same with the primer ' pLexA-TaHDT701-F '	For construct of pCAMBIA-YN-TaHDT701, F primer
pCAMBIA-YN-TaHDT701-R	same with the primer ' pLexA-TaHDT701-R '	For construct of pCAMBIA-YN-TaHDT701, R primer
pCAMBIA-YC-TaHDA6-F	5'GGGGACAAGTTTGTACAAAAAAGCAGGCTTCATGGCGGCGTCCGGCGAGGGAG3'	For construct of pCAMBIA-YC-TaHDA6, F primer
pCAMBIA-YC-TaHDA6-R	5'GGGGACCACTTTGTACAAGAAAGCTGGGTCCTACAAATCATCATTTGATG3'	For construct of pCAMBIA-YC-TaHDA6, R primer
pCAMBIA-YN-TaHOS15-F	5'GGGGACAAGTTTGTACAAAAAAGCAGGCTTCATGGGAGCGATAACCTCGG3'	For construct of pCAMBIA-YC-TaHOS15, F primer
pCAMBIA-YN-TaHOS15-R	5'GGGGACCACTTTGTACAAGAAAGCTGGGTCCTACATCCTGAAATCCATG3'	For construct of pCAMBIA-YC-TaHOS15, R primer
pCAMBIA-nLUC-TaHDT701-F	same with the primer ' pLexA-TaHDT701-F '	For construct of pCAMBIA-nLUC-TaHDT701, F primer
pCAMBIA-nLUC-TaHDT701-R	same with the primer ' pLexA-TaHDT701-R '	For construct of pCAMBIA-nLUC-TaHDT701, R primer
pCAMBIA-cLUC-TaHDA6-F	same with the primer ' pCAMBIA-YC-TaHDA6-F '	For construct of pCAMBIA-cLUC-TaHDA6, F primer
pCAMBIA-cLUC-TaHDA6-R	same with the primer ' pCAMBIA-YC-TaHDA6-R '	For construct of pCAMBIA-cLUC-TaHDA6, R primer
pCAMBIA-cLUC-TaHOS15-F	same with the primer ' pCAMBIA-YN-TaHOS15-F '	For construct of pCAMBIA-cLUC-TaHOS15, F primer
pCAMBIA-cLUC-TaHOS15-R	same with the primer ' pCAMBIA-YN-TaHOS15-F '	For construct of pCAMBIA-cLUC-TaHOS15, R primer
pGEX4T-TaHDT701-F	5'AGGGGGGATCCATGGAGTTCTGGGGCCTTGAG3'	For construct of pGEX4T-TaCHR729, F primer
pGEX4T-TaHDT701-R	5'AGCCCGAATTCCTACTTGGCCTCATGCTTGG3'	For construct of pGEX4T-TaCHR729, R primer
qRT-PCR-TaGAPDH-F	5' TTAGACTTGCGAAGCCAGCA 3'	qRT-PCR primer for internal control gene TaGAPDH, F primer
qRT-PCR-TaGAPDH-R	5' AAATGCCCTTGAGGTTTCCC 3'	qRT-PCR primer for internal control gene TaGAPDH, R primer
qRT-PCR-TaHDT701-F	5'AAGCCTAACCAGTCCGTCAAG3'	qRT-PCR assay for *TaHDT701*, F primer
qRT-PCR-TaHDT701-R	5' CTTGTAGCCAGAGAAGAAGAC 3'	qRT-PCR assay for *TaHDT701*, R primer
qRT-PCR-TaHDA6-F	5' CTTGAGTGATGGTATTGATG3'	qRT-PCR assay for *TaHDA6*, F primer
qRT-PCR-TaHDA6-R	5' TAACCTCCACCTCCCAAAAC3'	qRT-PCR assay for *TaHDA6*, R primer
qRT-PCR-TaHOS15-F	5' TTCTAGCTTCTGGGTCAGG3'	qRT-PCR assay for *TaHOS15*, F primer
qRT-PCR-TaHOS15-R	5' TGCTTGGCCATCATAGGAG3'	qRT-PCR assay for TaHOS15, R primer
qRT-PCR-TaPR1-F	5' GAGAATGCAGACGCCCAAGC 3'	qRT-PCR assay for *TaPR1*, F primer
qRT-PCR-TaPR1-R	5' CTGGAGCTTGCAGTCGTTGATC 3'	qRT-PCR assay for *TaPR1*, R primer
qRT-PCR-TaPR2-F	5' AGGATGTTGCTTCCATGTTTGCCG 3'	qRT-PCR assay for *TaPR2*, F primer

qRT-PCR-TaPR2-R	5' AAGTAGATGCGCATGCCGTTGATG 3'	qRT-PCR assay for *TaPR2*, R primer
qRT-PCR-TaPR5-F	5' CTTCTACATCAAGAACAACTG3'	qRT-PCR assay for *TaPR5*, F primer
qRT-PCR-TaPR5-R	5' CAGTCGCCGGTCTGGCAG3'	qRT-PCR assay for *TaPR5*, R primer
qRT-PCR-TaWRKY45-F	5' CTTCTCCCGCGCCATCCAC3'	qRT-PCR assay for *TaWRKY45*, F primer
qRT-PCR-TaWRKT45-R	5' CGTACTTGCGCCATGTCTG3'	qRT-PCR assay for *TaWRKY45*, R primer
qRT-PCR-TaEF1-F	5' CAGGACGTTTACAAGATTG3'	qRT-PCR assay for *TaEF1*, F primer
qRT-PCR-TaEF1-R	5' CAAAACCACGCTTCAGATC3'	qRT-PCR assay for *TaEF1*, R primer
ChIP-qPCR-TaPR1-A-P1-F	5'TTTCCTGAAGCTGGTATTG3'	ChIP-qPCR assay for TaPR1-A-P1, F primer
ChIP-qPCR-TaPR1-A-P1-R	5'CGGAAGCAACTGTATTGC3'	ChIP-qPCR assay for TaPR1-A-P1, R primer
ChIP-qPCR-TaPR1-A-P2-F	5'AGTGTTAGTAAGGTTGAGAG3'	ChIP-qPCR assay for TaPR1-A-P2, F primer
ChIP-qPCR-TaPR1-A-P2-R	5'GCATATGGTTCCAGGAGGAG3'	ChIP-qPCR assay for TaPR1-A-P2, R primer
ChIP-qPCR-TaPR1-B-P1-F	5'GGGCCTGTCAAATCAGAG3'	ChIP-qPCR assay for TaPR1-B-P1, F primer
ChIP-qPCR-TaPR1-B-P1-R	5'CAAATCAACCAATACCAG3'	ChIP-qPCR assay for TaPR1-B-P1, R primer
ChIP-qPCR-TaPR1-B-P2-F	5'ACAGAAAGTGACGTCCAC3'	ChIP-qPCR assay for TaPR1-B-P2, F primer
ChIP-qPCR-TaPR1-B-P2-R	5'ACTCATGGTTAATCTAC3'	ChIP-qPCR assay for TaPR1-B-P2, R primer
ChIP-qPCR-TaPR1-D-P1-F	5'ATCATTCCAAACAGAAAG3'	ChIP-qPCR assay for TaPR1-D-P1, F primer
ChIP-qPCR-TaPR1-D-P1-R	5'CCAACCCTCAATTTGTAC3'	ChIP-qPCR assay for TaPR1-D-P1, R primer
ChIP-qPCR-TaPR1-D-P2-F	5'AAAATTTACCATAGTAAG3'	ChIP-qPCR assay for TaPR1-D-P2, F primer
ChIP-qPCR-TaPR1-D-P2-R	5'TGGCTCACGTATGTAAC3'	ChIP-qPCR assay for TaPR1-D-P2, R primer
ChIP-qPCR-TaPR2-A-P1-F	5'CTAGTGACTTCGTCTAAG3'	ChIP-qPCR assay for TaPR2-A-P1, F primer
ChIP-qPCR-TaPR2-A-P1-R	5'TTTTTTGGGACTTTTTTAAC3'	ChIP-qPCR assay for TaPR2-A-P1, R primer
ChIP-qPCR-TaPR2-A-P2-F	5'GCAAACGGGAGTGGAACAG3'	ChIP-qPCR assay for TaPR2-A-P2, F primer
ChIP-qPCR-TaPR2-A-P2-R	5'GATGCATGGCGCGCGTCGTG3'	ChIP-qPCR assay for TaPR2-A-P2, R primer
ChIP-qPCR-TaPR2-B-P1-F	5'GGCCGTCAGCCCAGCGCG3'	ChIP-qPCR assay for TaPR2-B-P1, F primer
ChIP-qPCR-TaPR2-B-P1-R	5'GTAAAACAGAAAAGCAATC3'	ChIP-qPCR assay for TaPR2-B-P1, R primer
ChIP-qPCR-TaPR2-B-P2-F	5'TAGAACCTGTAGTACACTAG3'	ChIP-qPCR assay for TaPR2-B-P2, F primer
ChIP-qPCR-TaPR2-B-P2-R	5'CATGATAATGGACGTCACTC3'	ChIP-qPCR assay for TaPR2-B-P2, R primer
ChIP-qPCR-TaPR2-D-P1-F	5'GTTTTATACGTGTTTCACATG3'	ChIP-qPCR assay for TaPR2-D-P1, F primer
ChIP-qPCR-TaPR2-D-P1-R	5'GAAATCCATACGAAATATAC3'	ChIP-qPCR assay for TaPR2-D-P1, R primer
ChIP-qPCR-TaPR2-D-P2-F	5'TGAAGTGTGGGCTGAATG3'	ChIP-qPCR assay for TaPR2-D-P2, F primer
ChIP-qPCR-TaPR2-D-P2-R	5'CTTCCGCCTTTCTTCCACG3'	ChIP-qPCR assay for TaPR2-D-P2, R primer
ChIP-qPCR-TaPR5-A-P1-F	5'GTGCTTTATCAACAGTTAC3'	ChIP-qPCR assay for TaPR5-A-P1, F primer
ChIP-qPCR-TaPR5-A-P1-R	5'CTTTCACCATTGCGAGATAAC3'	ChIP-qPCR assay for TaPR5-A-P1, R primer
ChIP-qPCR-TaPR5-A-P2-F	5'TTAATCTTTAATAAAGCAG3'	ChIP-qPCR assay for TaPR5-A-P2, F primer
ChIP-qPCR-TaPR5-A-P2-R	5'GTATGACCAATGAAGCCTA3'	ChIP-qPCR assay for TaPR5-A-P2, R primer
ChIP-qPCR-TaPR5-B-P1-F	5'GATATTTAGCTTTTGAAAC3'	ChIP-qPCR assay for TaPR5-B-P1, F primer
ChIP-qPCR-TaPR5-B-P1-R	5'CAGAATTGGTTGGTGGGAC3'	ChIP-qPCR assay for TaPR5-B-P1, R primer
ChIP-qPCR-TaPR5-B-P2-F	5'ACCTCTTGGTTGTTCTTC3'	ChIP-qPCR assay for TaPR5-B-P2, F primer
ChIP-qPCR-TaPR5-B-P2-R	5'TACGTTGCATTTCGTGCA3'	ChIP-qPCR assay for TaPR5-B-P2, R primer
ChIP-qPCR-TaPR5-D-P1-F	5'ATAGCGAGTCCGGTTTTG3'	ChIP-qPCR assay for TaPR5-D-P1, F primer
ChIP-qPCR-TaPR5-D-P1-R	5'GTGATGACCTCACAAATC3'	ChIP-qPCR assay for TaPR5-D-P1, R primer
ChIP-qPCR-TaPR5-D-P2-F	5'ATGTATTTCCAGTCCACTTAG3'	ChIP-qPCR assay for TaPR5-D-P2, F primer
ChIP-qPCR-TaPR5-D-P2-R	5'GAAAAGTATGGCCAATGAAG3'	ChIP-qPCR assay for TaPR5-D-P2, R primer
ChIP-qPCR-TaWRKY45-A-P1-F	5'CTACACATGAATACCTCTG 3'	ChIP-qPCR assay for TaWRKY45-A-P1, F primer
ChIP-qPCR-TaWRKY45-A-P1-R	5'TATCCTTCTTCTCACCTTC3'	ChIP-qPCR assay for TaWRKY45-A-P1, R primer
ChIP-qPCR-TaWRKY45-A-P2-F	5'GATCAGGTGTGCCGTTGATGC3'	ChIP-qPCR assay for TaWRKY45-A-P2, F primer
ChIP-qPCR-TaWRKY45-A-P2-R	5'GGTGACGAGTGCGGGTCGTC3'	ChIP-qPCR assay for TaWRKY45-A-P2, R primer

ChIP-qPCR-TaWRKY45-B-P1-F	5'GCATTAATTTGGGCTATATG3'	ChIP-qPCR assay for TaWRKY45-B-P1, F primer
ChIP-qPCR-TaWRKY45-B-P1-R	5'CAAACCAGTTGTAAGTATTTC3'	ChIP-qPCR assay for TaWRKY45-B-P1, R primer
ChIP-qPCR-TaWRKY45-B-P2-F	5'TGACTTCACGTCACCATGGATC3'	ChIP-qPCR assay for TaWRKY45-B-P2, F primer
ChIP-qPCR-TaWRKY45-B-P2-R	5'GGTCGTCAGCGGATGACGAG3'	ChIP-qPCR assay for TaWRKY45-B-P2, R primer
ChIP-qPCR-TaWRKY45-D-P1-F	5'CTTGTACGTAGATTAATG3'	ChIP-qPCR assay for TaWRKY45-D-P1, F primer
ChIP-qPCR-TaWRKY45-D-P1-R	5'CAAGTTGTCCTTCTTCATTG 3'	ChIP-qPCR assay for TaWRKY45-D-P1, R primer
ChIP-qPCR-TaWRKY45-D-P2-F	5'TCCAACAATAGGATAGCTGG3'	ChIP-qPCR assay for TaWRKY45-D-P2, F primer
ChIP-qPCR-TaWRKY45-D-P2-R	5'CACGTCACCCTGCATCAAC3'	ChIP-qPCR assay for TaWRKY45-D-P2, R primer
ChIP-qPCR-TaEF1-A-P1-F	5'GCAATGTCCAAACACGCCA3'	ChIP-qPCR assay for TaEF1-A-P1, F primer
ChIP-qPCR-TaEF1-A-P1-R	5'GAAGAAGAAGAACGAAC3'	ChIP-qPCR assay for TaEF1-A-P1, R primer
